# Cirrhotic cardiomyopathy: a subject that's always topical

**DOI:** 10.2144/fsoa-2023-0110

**Published:** 2024-05-15

**Authors:** Mona Boudabbous, Rania Hammemi, Hela Gdoura, Lassad Chtourou, Manel Moalla, Leila Mnif, Ali Amouri, Leila Abid, Nabil Tahri

**Affiliations:** 1 Gastroenterology Department, Hédi Chaker Hospital, Sfax, Tunisia; 2 Cardiology, Department, Hédi Chaker Hospital, Sfax, Tunisia; 3 Medecin sfax university, Sfax university, Tunisia

**Keywords:** cirrhosis, cirrhotic cardiomyopathy, diastolic dysfunction, systolic dysfunction

## Abstract

Cirrhosis is the final stage in the development of hepatic fibrosis in chronic liver disease. It is associated with major hemodynamic disturbances defining the hyperdynamic circulation and may be complicated by specific cardiac involvement or cirrhotic cardiomyopathy which is a silent clinical condition that typically remains asymptomatic until the late stages of liver disease.

Recently, new criteria defining CC, based on modern concepts and knowledge of heart failure, have been proposed. Despite knowledge of the main mechanisms behind this entity, there is no specific treatment available for cirrhotic cardiomyopathy. The management approach for symptomatic cardiomyopathy in cirrhotic patients is similar to that for left ventricular failure in non-cirrhotic individuals.

## Abstract (plain language summary)

The exact prevalence of cirrhotic cardiomyopathy (CC) is unknown, due to the latent nature of the disease. The most recent studies based on echocardiographic criteria found an incidence of 23%.

Recently, new criteria defining CC have been proposed. They are based on modern concepts and knowledge of heart failure.

The main pathogenic mechanisms of cirrhotic cardiomyopathy (CC) include:

- Impaired signal transduction of β-receptors.
- Activation of negative inotropic pathways.
- Dysfunction of potassium and calcium ion channels.
- Alterations in the plasma membrane structure.

The electrophysiological alterations most frequently observed in cirrhotic are QT interval prolongation, chronotropic incompetence and electromechanical dissociation.

Cardiac histological lesions combine myocardial and subendothelial hypertrophy and edema. These abnormalities were more frequently found in cases of decompensated cirrhosis.

cirrhotic cardiomyopathy (CC) is a silent clinical condition that typically remains asymptomatic until the late stage of liver disease.

Incorporating 2D-STE into the evaluation allows for a more comprehensive assessment of systolic function beyond the traditional measures like EF.

Currently, there is no specific treatment available for cirrhotic cardiomyopathy. The management approach for symptomatic cardiomyopathy in cirrhotic patients is similar to that for left ventricular failure in non-cirrhotic individuals.

Cirrhosis represents the ultimate phase of hepatic fibrosis progression in cases of chronic liver disease. This condition is characterized by significant hemodynamic disruptions that define a hyperdynamic circulation. Additionally, cirrhosis can potentially give rise to specific cardiac complications or a condition known as cirrhotic cardiomyopathy (CC) [1]. CC encompasses various abnormalities in morphology, function, electrophysiology, and biochemistry observed in individuals with cirrhosis who do not have any other recognized cardiac pathologies [2].

The aim of this review is to clarify the epidemiological and clinical features, pathophysiological mechanisms and principles of therapeutic management of this entity.

## Epidemiology

The prevalence of cirrhotic cardiomyopathy is unknown, due to the latent nature of the disease, which only appears when the patient is under stress. However, it is estimated that half of patients scheduled for liver transplantation show signs of cardiac dysfunction, with post-operative mortality from heart failure ranging from 7% to 21% [3]. Nevertheless, in a series published in 2013, Enache *et al.* observed an incidence of 23.4% on echocardiographic criteria [4].

## Definition

During the 2005 World Congress of Gastroenterology in Montreal, a panel of experts put forward preliminary diagnostic criteria for CC (Table 1). However, advancements in our understanding of ventricular dysfunction, particularly in the past decade, have made these criteria outdated [5]. As a result, a group of multidisciplinary experts, including hepatologists, anesthetists and cardiologists, convened to propose updated criteria based on modern concepts and knowledge of heart failure.

**Table 1.** World Congress of Gastroenterology in Montreal in 2005's criteria defining cirrhotic cardiomyopathy.

Diagnostic criteria: – Abnormal contractile systolic response to stress – Diastolic dysfunction at rest – Absence of significant cardiopulmonary abnormality
Systolic dysfunction: at least one of the following two criteria: – Increase in cardiac output attenuated by exercise, or in the event of volemic or pharmacological stress – Resting ejection fraction <50%. Absence of signs of heart failure at rest
Diastolic dysfunction – E/A <1 (age corrected) – E wave deceleration time >200 ms – Prolonged isovolumic relaxation time >80 ms
Additional criteria: – Electrophysiological abnormalities – Abnormal chronotropic response – Prolongation of QTc interval – Autonomic dysfunction – Enlarged left atrium – Increased myocardial mass and LV wall thickness – Increased pro BNP, BNP, ANP and troponin – QT interval prolongation

ANP: Atrial natriuretic peptide; A: Wave A (atrial); BNP: Brain natriuretic peptide; E: Wave E (early); LV: Left ventricle.

The quantification of left ventricular systolic function through LVEF assessment has limitations in patients with cirrhosis. Additionally, evaluating the altered contractile response during exercise testing is often challenging in these patients. Firstly, pharmacological beta-blockade is common in this patient population. Secondly, the definition of impaired cardiac functional reserve has expanded to include hemodynamic changes, other measures of contractile function, as well as impaired diastolic reserve [6,7]. Moreover, the vasodilatory state associated with ESLD leads to a decrease in afterload and, consequently, a normal or even increased LVEF. Therefore, while LVEF remains an important measure of overall systolic function, additional indicators are particularly necessary in cirrhotic patients to assess cardiac contractility.

Echocardiographic strain imaging, also known as myocardial strain imaging, has emerged as an objective method to quantify regional myocardial contractile function. Strain can be categorized into circumferential, longitudinal, radial and transverse strain, allowing for a more comprehensive evaluation of contractile function beyond LVEF, which primarily reflects radial function. As longitudinal contractile function is often impaired prior to the loss of radial function, global longitudinal strain (GLS) can identify myocardial contractile dysfunction in individuals with preserved LVEF across different populations [^8-10^].

GLS represents longitudinal myocardial shortening as a percentage, indicating the change in length during systole relative to the baseline length in diastole. Normally, myocardium shortens in the longitudinal plane during systole, so GLS is typically expressed as a negative number. Guidelines from the American Society of Echocardiography (ASE) and the European Association of Cardiovascular Imaging (EACVI) define a GLS of less than -16% as abnormal, a GLS of -18% or more (plus negative) as normal, and a GLS of -16% to -18% as borderline in adults. To avoid confusion among healthcare providers, changes in deformation are described in absolute values [^11-13^].

Although assessment of longitudinal strain is influenced by age, gender, and left ventricular loading conditions, the ASE recommended its use as an adjunct to evaluate LV systolic function in patients with normal LVEF in 2015. However, data on strain imaging for detecting CCM in patients with normal LVEF are limited and contradictory. Some studies have shown normal longitudinal strain in cirrhotic patients, while others, including a multicenter study, have demonstrated diminished longitudinal strain in patients with cirrhosis [^14-16^].

**Figure 1.**
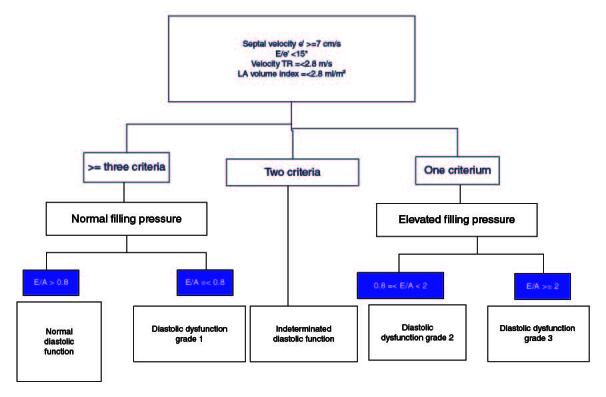
New criteria defining cirrhotic cardiomyopathy. A: Atrial wave; E: Early wave; LA: Left Atrial TR: Tricuspid regurgitation.

Recently, new criteria defining CC have been proposed [1]. For systolic function, a reduced LVEF or LMS in the absence of known cardiac disease defines CC. Regarding diastolic function, these authors have endorsed the ASE/EACV recommendations with minor modifications (Figure 1).

## Physiopathology

**Figure 2.**
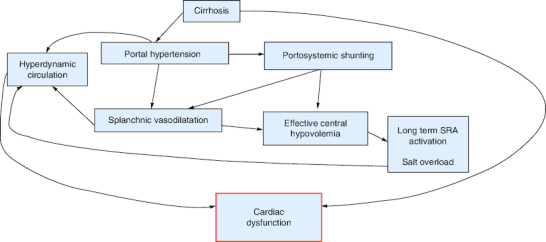
Physiopathology of cirrhotic cardiomyopathy. RAS: Renin angiotensin system.

The primary hemodynamic disturbance in portal hypertension (PH) is splanchnic vasodilation, which leads to a decrease in systemic vascular resistance, blood pressure and central blood volume, creating a state of effective hypovolemia (Figure 2). Compensatory mechanisms, such as the central nervous system and the renin-angiotensin system, are activated to increase heart rate (HR) and cardiac output (CO) to ensure adequate peripheral perfusion, resulting in the characteristic ‘hyperdynamic circulation’ observed in cirrhosis. This hyperdynamic state is characterized by increased cardiac output, stroke volume, and heart rate. As the disease progresses, plasma volume increases due to activation of the renin-angiotensin-aldosterone system (RAAS), the sympathetic nervous system (SNS), and release of antidiuretic hormone (ADH) [5].

Despite the increase in plasma volume, an imbalance persists between central and arterial vascular volume on one hand and splanchnic volume on the other, leading to ongoing effective hypovolemia [5,17]. This redistribution sets up a vicious cycle in which central hypovolemia activates additional baroreceptors in the SNS and RAAS, resulting in fluid retention and redistribution. Over time, this cascade affects the structure and function of cardiac muscle, leading to systolic and diastolic dysfunction and electrophysiological abnormalities [18]. Overall, there is a direct relationship between vasodilation, systemic circulatory dysregulation, the progression of liver disease, the development of complications, and prognosis. However, assessing systemic hemodynamic changes in clinical practice is challenging. Patients with early-stage cirrhosis may exhibit circulatory hyperkinesia, while some individuals with decompensated cirrhosis and significant fluid retention may present with relatively normal circulation [17]. Moreover, studies have shown that patients with advanced disease and refractory ascites may have low cardiac output [19,20]. Additionally, the use of β-blockers can attenuate circulatory hyperkinesia [21].

The main pathogenic mechanisms of cirrhotic cardiomyopathy (CC) include:

- Impaired signal transduction of β-receptors, resulting in dysfunctional sympathetic control, including desensitization of cardiac chronotropic and inotropic responses;
- Activation of negative inotropic pathways due to substances passing from the splanchnic circulation to the systemic circulation, such as nitric oxide (NO), carbon dioxide (CO), endocannabinoids and tumor necrosis factor (TNF), leading to impaired myocardial contractile function and altered β-adrenergic contractile response;
- Dysfunction of potassium and calcium ion channels due to the hyperdynamic circulation;
- Alterations in the plasma membrane structure.

## Electrophysiological alterations

The electrophysiological alterations most frequently observed in cirrhotics are QT interval prolongation, chronotropic incompetence and electromechanical dissociation.

### QT interval prolongation

QT prolongation is a common abnormality in advanced cirrhosis, with a frequency exceeding 60% [22]. The exact pathogenesis of QT prolongation in cirrhosis is not yet fully understood, but it appears to be associated with the presence of portal hypertension and porto-systemic shunts. Importantly, this electrical abnormality is not related to the specific etiology of liver disease [22,23]. After liver transplantation, the occurrence of QT prolongation is considered a poor prognostic factor [24]. QT prolongation is characterized by abnormal myocardial repolarization, posing a risk of torsades de pointes, ventricular arrhythmias and sudden death, although such events are rare in cirrhotic patients. However, in the context of gastrointestinal hemorrhage, QT prolongation is associated with reduced survival [25].

Studies by M Bernardi *et al.* have demonstrated a close correlation between QT prolongation, the severity of hepatocellular failure, elevated circulating plasma norepinephrine levels, and reduced survival [22]. J Henriksen *et al.* proposed that the effect of beta-blockers on the QT interval may be mediated through a vagal-type mechanism [26]. A Zambruni *et al.* found that beta-blockers can reduce QT prolongation but only in patients who already had a prolonged QT interval before treatment [27]. Conversely, beta-blockers may be associated with increased mortality in cirrhotic patients with refractory ascites [28]. Furthermore, these drugs may increase the risk of circulatory dysfunction [21,29]. It is also important to exercise caution when using other medications known to cause QT prolongation, such as quinolones, macrolides and drugs affecting intestinal motility. In such cases, monitoring is advisable [30].

### Chronotropic incompetence

This is the inability of the heart to respond to a physiological or pharmacological stress stimulus with an appropriate rise in heart rate [31]. The pathogenesis of this problem is poorly understood. Alterations in the β-adrenergic receptor have been suggested in the face of a greater need for β-agonists [27].

### Electromechanical asynchronization

Defined as the delay between the excitation and contraction phases, it is assessed by the systolic interval time or by simultaneous measurement of the aortic pressure curve and an ECG trace [32]. This delay increases with QT. This anomaly is thought to be linked to a defect in the β-adrenergic receptor, or post-receptor [26]. Its clinical impact has not been identified.

## Cardiac histological lesions

CC lesions combine myocardial and subendothelial hypertrophy and edema. In 1176 autopsies performed over a 12-year period, macroscopic cardiac abnormalities were found in 135 cirrhotics, in particular left ventricular hypertrophy in 47% of cases of alcoholic cirrhosis and 41% of cases of cirrhosis of other etiologies. These abnormalities were more frequently found in cases of decompensated cirrhosis (62%) [33].

## Diagnosis

As mentioned earlier, cirrhotic cardiomyopathy (CC) is a silent clinical condition that typically remains asymptomatic until the late stage of liver disease. Therefore, it is crucial to regularly evaluate patients with cirrhosis for any abnormalities, as they often present no symptoms until a stressor occurs. Various studies have demonstrated that procedures such as *trans*-jugular intrahepatic portosystemic shunt (TIPS) placement and liver transplantation can lead to sudden heart failure, cardiovascular events and postoperative pulmonary edema if undetected diastolic and systolic dysfunction is present before the procedure [34]. When assessing systolic dysfunction, clinicians should not solely rely on conventional echocardiographic evaluation of function, which measures the ejection fraction (EF) at rest. In cirrhotic patients, systolic function often appears normal due to the hyperdynamic state. Currently, 2D speckle tracking echocardiography (2D-STE) has been proposed as an additional tool for accurate assessment of systolic function. This method can identify subclinical left ventricular valve dysfunction at an earlier stage [35]. Therefore, incorporating 2D-STE into the evaluation allows for a more comprehensive assessment of systolic function beyond the traditional measures like EF

## Treatement

Currently, there is no specific treatment available for cirrhotic cardiomyopathy. The management approach for symptomatic cardiomyopathy in cirrhotic patients is similar to that for left ventricular failure in non-cirrhotic individuals.

The recommended measures include rest, oxygen therapy with ventilation if necessary, and a low-salt diet. Diuretics play a crucial role in the medical treatment of cirrhotic cardiomyopathy [36]. Non-selective beta-blockers can help reduce the hyperdynamic load in cirrhotic patients and improve the QT interval. However, it is still unclear whether the shortening of the QT interval has a beneficial effect on prognosis [37]. It should be noted that the use of beta-blockers may increase mortality in patients with refractory ascites [38]. Similarly, angiotensin-converting enzyme (ACE) inhibitors are not recommended as they can worsen the already present vasodilation characteristic of advanced cirrhosis [39].

For patients with heart failure in NYHA class III and/or IV, the addition of an aldosterone antagonist to their treatment regimen may provide potential benefits. Studies have demonstrated a significant reduction in hospitalization and mortality rates by 35% and 30%, respectively, with the use of aldosterone antagonists. Aldosterone promotes myocardial fibrosis, sympathetic nervous system (SNS) activation and baroreceptor dysfunction [40]. However, further research is needed to fully understand the role of aldosterone antagonists in cirrhotic cardiomyopathy.

In addition, there are ongoing investigations into new pharmacological agents targeting inflammatory cytokines and nitric oxide (NO) for the treatment of cirrhotic cardiomyopathy. Examples include farnesoid X receptor agonists, which are involved in the intrahepatic production of vasodilatory hydrogen sulfide, and NCX-1000, a novel agent that releases NO in the liver.

Lastly, liver transplantation leads to the reversibility of both cirrhotic cardiomyopathy and the hyperdynamic state within 6–12 months [41,42].

## Conclusion

The prevalence of cirrhotic cardiomyopathy is unknown, but it is estimated that half of patients requiring liver transplantation show signs of cardiac dysfunction. It is an indolent clinical entity that remains silent until the terminal stage of liver disease. Recently, new criteria defining cirrhotic cardiomyopathy have been proposed. The disease results from an imbalance between central and arterial vascular volume on the one hand and splanchnic volume on the other, which maintains effective hypovolaemia.

In the long term, this cascade affects the structure and function of the heart muscle, leading to systolic and diastolic dysfunction and electrophysiological abnormalities. Cardiac histological lesions combine hypertrophy and myocardial and underlying endothelial oedema. The electrophysiological changes most frequently observed in cirrhotics are QT interval prolongation, chronotropic incompetence and electromechanical dissociation. To date, there is no specific treatment for cirrhotic cardiomyopathy. The measures to be recommended in the case of symptomatic cardiomyopathy are similar to those to be taken in the case of left ventricular failure in a non-cirrhotic patient, namely. These include rest, oxygen therapy with ventilation if necessary, and a low-salt diet.

## Future perspective

Prospective multicenter studies with sufficient cohort follow-up and control of bias's causes will provide a clearer picture of the prognosis for this entity and will help to identify the most appropriate treatment.
